# Healthcare Priority-Setting: Chat-Ting Is Not Enough: Comment on "Swiss-CHAT: Citizens Discuss Priorities for Swiss Health Insurance Coverage"

**DOI:** 10.15171/ijhpm.2018.66

**Published:** 2018-07-28

**Authors:** Leonard M. Fleck

**Affiliations:** Center for Ethics, College of Human Medicine, Michigan State University, East Lansing, MI, USA.

**Keywords:** Rationing, Priority-Setting, Rational Democratic Deliberation, Healthcare Justice, Cost-Effectiveness

## Abstract

CHAT has its limits. It is a three-hour exercise. However, the real world problems of healthcare rationing and priority-setting are too complex for a three-hour exercise. What is needed, as a supplement, are sustained processes of rational democratic deliberation that can address the challenges to healthcare justice posed by costly emerging medical technologies, such as these targeted cancer therapies.

The CHAT game is a lot of fun. It is engaging, thought-provoking, and thoughtfully designed. However, healthcare priority-setting is too complex for just a CHAT. In this commentary, my goal is to identify the limits of CHAT in order to make clear the need to move beyond CHAT. I will discuss three claims. First, in the real world, just healthcare rationing and priority-setting require sustained rational democratic deliberation about very complex, concrete trade-offs, not just abstract principles. Second, what I refer to as the “liberalism problem” also requires sustained public deliberation. That phrase refers to the question of whether socially controversial medical procedures, such as pre-implantation genetic diagnosis, should be publicly funded. Third, our “sense of justice” is complex and very much context-dependent, which again requires sustained public deliberation to fashion a considered judgment of healthcare justice specific to some concrete rationing problem.

The CHAT game asks participants to set priorities among broad categories of healthcare interventions and values. To my mind, that has *limited* social value. Hurst et al report, for example, that 66% of participants endorsed “greater adherence to cost-effectiveness guidelines in chronic care.”^1^ Given concerns about escalating healthcare costs, broad endorsement of that guideline is not surprising. However, most of the targeted cancer therapies and immunotherapies used to treat metastatic cancers are not cost-effective (using $100 000 per quality-adjusted life year [QALY] as the threshold) nor do they yield clinically meaningful benefit (though we note that no public deliberative process or legislative policy in Switzerland has specifically endorsed any such number, whether higher or lower).^2,3^ On average, most of these targeted therapies yield survival gains measurable in months, not years, with costs ranging from $100 000 for a course of treatment to more than $500 000 (for CAR T-cell therapy for B-cell lymphoma).^4^ In one study only 13 of 37 of these targeted cancer therapies met the threshold of the European Society of Medical Oncology for clinically meaningful benefit.^5^ Should we conclude from that research that Swiss CHAT participants would give up socially funded access to all these cancer therapies that were not cost-effective and did not yield clinically meaningful benefit? This clearly needs to be known, at least as far as social policy is concerned.

In the United Kingdom, the National Institute for Clinical Excellence (NICE) is responsible for determining whether or not these targeted cancer therapies will be funded by the National Health Service (NHS), which has a fixed annual budget, unlike Switzerland and many other countries, including the United States. NICE uses cost-effectiveness analysis in their judgments, though that is not exclusively determinative. Consequently, many of these cancer therapies are included, but a substantial number are excluded as well. NICE is entirely independent of the British government, which gives it immunity from political pressure. However, political pressure did force the British government to create a Cancer Drugs Fund to provide access to those drugs not approved by NICE. Over the period 2010-2016 that fund expended £1.27 billion for almost 100 000 cancer patients without gathering any data on outcomes for those patients (perhaps because the data would have been embarrassing and only confirm NICE’s original judgment).^6^ That political pressure is described as being “populist” in origin, which might reflect something “democratic,” though unlikely “deliberatively democratic.” Perhaps a sustained deliberative process over weeks or months by a representative group of British citizens would have yielded the same outcome, though the discussion below provides reasons for thinking otherwise.

Here are some concrete questions related to these cancer drugs that must be answered. Some patients with metastatic lung cancer might be taking one of these $100 000 cancer drugs for a year but gain only three additional months of life. That yields an incremental cost-effectiveness value of $400 000 per QALY. Is that a good or just use of limited social resources? Could other healthcare needs have been met that would have yielded more health good for more persons at a lower cost per QALY? Should we ignore that question because these lung cancer patients are faced with a terminal outcome in the near future? However, 80% of Swiss CHAT participants wanted to exclude from coverage “invasive life-sustaining measures in dying patients.” What should we imagine those participants regarded as the scope of that directive? Metastatic cancer is a terminal illness, though these patients are not *imminently* dying. Many of these cancer drugs are given by infusion. Is that “invasive enough” to invoke the above directive?

A small percentage of these cancer patients are described as “super responders” to these drugs. They gain extra years of life, though each of those years will cost $100 000 or more. At present, physicians have no idea before the fact who those patients might be. That means treatment for the vast majority of these metastatic cancer patients will not be cost-effective, though it would be cost-effective for the 3% or 5% who might be super responders. Given those facts, what is the just choice to make from a social policy perspective? Should all these cancer patients have assured access to these drugs, though not cost-effective, perhaps based on an expanded sense of compassion? Alternatively, should cost-effectiveness considerations exclude *all* those cancer patients, thereby sacrificing the many extra years of life that could be gained by the super responders?

If this latter alternative seems harsh and insensitive, and if we consequently choose to provide these drugs to any cancer patients who might benefit to any degree, then what would morally justify restricting this expanded sense of just compassion to cancer patients alone? Would we not be equally ethically obligated to provide $250 000 left ventricular assist devices to patients in late-stage heart failure so that they might gain an extra year of life (sometimes more)? Likewise, if HIV patients have failed four-drug combinations that cost $35 000 per year, must we then provide them with the latest HIV drug that costs $118 000 per year?^7^ The list of chronic degenerative conditions in advanced stages that will result in death can be quite long, and will get longer with ongoing costly advances in medical technologies. Where should we draw the line?

To further complicate matters, research is occurring aimed at identifying biomarkers for these targeted cancer therapies that will distinguish patients most likely to have a strong response to these therapies (gain more than a year of life) from those likely to have only a marginal response. Assuming such research is successful, would Swiss CHAT participants, having endorsed greater attention to cost-effectiveness, also endorse restricting access to these cancer therapies to the likely strong responders? However, Hurst et al reported that “more participants agreed that benefits should be the same for everyone,” as opposed to restricting that expectation only to “really essential benefits.” If benefits are “the same for everyone,” does that imply that effectiveness and cost-effectiveness in relation to the clinical circumstances of individual patients are irrelevant? In other words, we would have to be willing to spend a $100 000 for an extra month of life or an extra year of life for the same therapy for the same cancer. Alternatively, should all of these advanced cancer therapies be regarded as being outside “really essential benefits,” the implication being that access to these therapies would depend upon individual ability to pay? However, that would appear to be a clear example of rationing, which 19% of CHAT participants say they would reject.

We can assume broad agreement with the proposition that the healthcare needs of cancer patients require a just and compassionate social response. As the discussion above indicates, translating that broad agreement into specific, costly, practical therapeutic choices is exceedingly complex (and not within the scope of what CHAT can accomplish). Adding to the complexity is the “liberalism problem.” A liberal, pluralistic society requires mutual respect for a broad diversity of reasonable values with diverse prioritizations among those values. What does that mean in practice when advocates for physician aid-in-dying request that this be a socially funded service available to metastatic cancer patients? Individuals with strong religious beliefs of a certain sort will vigorously object to paying taxes to support a practice they regard as profoundly morally objectionable. What does “mutual respect” require in that situation? Likewise, if a couple wishes to have children that are genetically their own, but they are both carriers of a cystic fibrosis mutation, they have the medical option of preimplantation genetic diagnosis (PGD), which has a cost of about $40 000 in the United States. The procedure itself involves the creation of multiple embryos grown *in vitro* to the 8-cell stage, when they would be genetically analyzed. Embryos free of some specific genetic mutation would be available for implantation; the rest would be discarded. Should access to PGD be socially funded for all future possible parents at risk of having a child with a genetic disorder that would very adversely affect the length of life or quality of life for that future possible child? This certainly seems like a worthy social objective, though again many individuals with specific religious commitments would vigorously object to paying taxes for a procedure to which they deeply conscientiously objected. What sort of social policy would reasonably respect the competing social values at stake in this situation? Medicine today is peppered with novel procedures and technologies that generate deep social divisiveness related to conflicting value commitments. A CHAT exercise cannot effectively address those social conflicts.

Philosophers tend to be advocates for one or another conception of justice: egalitarianism, utilitarianism, prioritarianism, libertarianism, sufficientarianism, or luck egalitarianism. However, if we were to review a broad range of widely socially endorsed considered judgments of healthcare justice, we would find all of those conceptions of justice reflected therein. Everyone with pneumonia or an inflamed appendix at risk of bursting has a just claim to needed medical care. Both egalitarians and utilitarians readily endorse this claim because the needed care is costworthy and effective. It is also very basic, and hence, endorsed by sufficientarians. Prioritarians are concerned about the just claims to needed care for those who are least well off health-wise. Compassion motivates endorsing those prioritarian concerns, at least for very ill patients for whom there is a reasonable chance of medical success, even if very costly. Some patients need extraordinarily expensive care for which chances of medical success are very marginal. Consequently, there will be broad social agreement that it is not unjust to allow individual ability to pay to determine access to such care, as libertarians would argue. However, what does healthcare justice require when an individual with metastatic lung cancer related to smoking needs $500 000 worth of cancer treatment for an extra year of life? What if genetic aspects of that individual made him more susceptible to lung cancer, unbeknownst to himself? What if he tried multiple times to quit smoking with some years of success? Is that a justice-relevant consideration? What if he had Whole Genome Sequencing ten years earlier and was duly advised of his genetic vulnerability but continued to smoke? Is that fact justice-relevant? A just and compassionate social response to such complex circumstances cannot emerge from a CHAT exercise.

The conclusion I wish to draw from all these questions is that a three-hour CHAT session cannot possibly yield thoughtful, stable, just agreement regarding the medically and morally complex issues identified above. This requires sustained rational democratic deliberation. For the interested reader I have written at length about what such a deliberative process must look like to be both just and legitimate.^8^ Space does not permit an extended description of that process. In general, when we have clear and widely shared agreement that a specific principle of justice yields a just rationing or allocation protocol, we have no need for a deliberative process. However, when there are conflicting or ambiguous intuitions of healthcare justice regarding novel therapeutic interventions, such as the targeted cancer therapies above, then we need a sustained fair process of democratic deliberation to construct a reasonable and “just enough” social response, what John Rawls refers to as “fair terms of cooperation.”^9^ This is how we revise and construct *public reason* in a liberal, pluralistic, democratic society that must function with shared understandings of healthcare justice.

To conclude on a more positive note, the virtue of CHAT is that it can serve as an effective motivator for these more sustained processes of democratic deliberation. The Swiss have a well-entrenched process of direct democracy, which would be a useful basis for the deliberative practices endorsed in this essay. However, in my judgment, these deliberative practices in Switzerland (and elsewhere) would need to be mediated and legitimated by a NICE-like entity, as in the United Kingdom, to formulate and authorize detailed policies. As I hope I have suggested clearly, the rationing and priority-setting decisions in healthcare today are too numerous and too complex for a feasible process of simple direct democracy.

## Ethical issues

Not applicable.

## Competing interests

Author declares that he has no competing interests.

## Author’s contribution

LMF is the single author of the paper.

## References

[1] Hurst SA, Schindler M, Goold SD, Danis M (2018). Swiss-CHAT: citizens discuss priorities for Swiss health insurance coverage. Int J Health Policy Manag.

[2] Davis C, Naci H, Gurpinar E, Poplavska E, Pinto A, Aggarwal A (2017). Availability of evidence of benefits on overall survival and quality of life of cancer drugs approved by European Medicine Agency: retrospective cohort study of drug approvals 2009-2013. BMJ.

[3] Schilsky RL, Schnipper LE (2018). Hans Christian Andersen and the value of new cancer treatments. J Natl Cancer Inst.

[4] Bach PB, Giralt SA, Saltz LB (2017). FDA approval of tisagenlecleucel: promise and complexities of a $475000 cancer drug. JAMA.

[5] Vivot A, Jacot J, Zeitoun JD, Ravaud P, Crequit P, Porcher R (2017). Clinical benefit, price, approval characteristics of FDA-approved new drugs for treating advanced solid cancer, 2000-2015. Ann Oncol.

[6] Triggle N. Cancer drugs fund ‘huge waste of money.’ BBC News. April 28, 2017. http://www.bbc.com/news/health-39711137. Accessed May 17, 2018.

[7] Blank C. FDA approves breakthrough drug for HIV patients with dwindling options. Modern Medicine Network: Formulary Watch. March 13, 2018. http://www.formularywatch.com/feature-articles/fda-approves-breakthrough-drug-hiv-patients-dwindling-options.

[8] Fleck LM. Just Caring: Health Care Rationing and Democratic Deliberation. New York: Oxford University Press; 2009.

[9] Rawls J. Political Liberalism. New York: Columbia University Press; 1996.

